# Evaluation of 2’-Fucosyllactose and *Bifidobacterium longum* Subspecies *infantis* on Growth, Organ Weights, and Intestinal Development of Piglets

**DOI:** 10.3390/nu14010199

**Published:** 2021-12-31

**Authors:** Victoria C. Daniels, Marcia H. Monaco, Mei Wang, Johanna Hirvonen, Henrik Max Jensen, Arthur C. Ouwehand, Ratna Mukherjea, Ryan N. Dilger, Sharon M. Donovan

**Affiliations:** 1 Division of Nutritional Sciences, University of Illinois, Urbana, IL 61801, USA; vcdanie2@illinois.edu (V.C.D.); rdilger2@illinois.edu (R.N.D.); 2 Department of Food Science and Human Nutrition, University of Illinois, Urbana, IL 61801, USA; monaco@illinois.edu (M.H.M.); meiwang@illinois.edu (M.W.); 3 IFF Health and Biosciences, 02460 Kantvik, Finland; Johanna.Hirvonen@iff.com (J.H.); Arthur.Ouwehand@iff.com (A.C.O.); 4 IFF R&D—Enabling Technologies, Advanced Analytical, 8220 Brabrand, Denmark; Henrik.Max.Jensen@iff.com; 5 IFF Health and Biosciences, Saint Louis, MO 63110, USA; rmukherjea@bensonhill.com; 6 Department of Animal Sciences, University of Illinois, Urbana, IL 61801, USA

**Keywords:** human milk oligosaccharides, 2’-fucosyllactose, *B infantis* Bi-26, intestine

## Abstract

Human milk is rich in oligosaccharides that influence intestinal development and serve as prebiotics for the infant gut microbiota. Probiotics and 2’-fucosyllactose (2’-FL) added individually to infant formula have been shown to influence infant development, but less is known about the effects of their synbiotic administration. Herein, the impact of formula supplementation with 2’-fucosyllactose (2’-FL) and *Bifidobacterium longum* subsp. *infantis* Bi-26 (Bi-26), or 2’-FL + Bi-26 on weight gain, organ weights, and intestinal development in piglets was investigated. Two-day-old piglets (*n* = 53) were randomized in a 2 × 2 design to be fed a commercial milk replacer ad libitum without (CON) or with 1.0 g/L 2’-FL. Piglets in each diet were further randomized to receive either glycerol stock alone or Bi-26 (10^9^ CFU) orally once daily. Body weights and food intake were monitored from postnatal day (PND) 2 to 33/34. On PND 34/35, animals were euthanized and intestine, liver and brain weights were assessed. Intestinal samples were collected for morphological analyses and measurement of disaccharidase activity. Dry matter of cecum and colon contents and *Bifidobacterium longum* subsp. *infantis* abundance by RT-PCR were also measured. All diets were well tolerated, and formula intake did not differ among the treatment groups. Daily body weights were affected by 2’-FL, Bi-26, and day, but no interaction was observed. There was a trend (*p* = 0.075) for greater total body weight gain in CON versus all other groups. Jejunal and ascending colon histomorphology were unaffected by treatment; however, there were main effects of 2’-FL to increase (*p* = 0.040) and Bi-26 to decrease (*p* = 0.001) ileal crypt depth. The addition of 2’-FL and/or Bi-26 to milk replacer supported piglet growth with no detrimental effects on body and organ weights, or intestinal structure and function.

## 1. Introduction

The postnatal period is a critical time for gastrointestinal and immune development, which are stimulated by bioactive components in human milk (HM) and the gut microbiota [1]. The bioactive components in HM include among others the prebiotic human milk oligosaccharides (HMOs) [2,3] and the milk microbiota [4]. The HMOs are the third most abundant solid component of HM after lipid and lactose, but their concentrations are influenced by maternal genetics, stage of lactation, seasonality and geographic location [5]. 2’-fucosyllactose (2’-FL), a neutral 2-linked fucosylated oligosaccharide [4], is one of the predominant HMO, but its synthesis is influenced by maternal secretor status [5]. 2’-FL concentration averages 2–3 g/L in HM of secretor mothers, but is undetectable in the milk of non-secretors [5]. 

HMOs resist gastrointestinal digestion [6,7], allowing intact HMO to influence various aspects of infant development. In the intestine, 2’-FL dose-dependently decreased cell proliferation and increased sucrase activity in cultured cells [8,9], suggesting a shift towards differentiation. In vivo, dietary HMO increased cecal barrier function in mice [10]. In the colon, HMOs are metabolized by the microbiota, and some species of *Bifidobacterium* and *Bacteroides* utilize HMOs as their primary energy source [11]. 

*Bifidobacterium* species predominate in fecal samples of breastfed infants due in part to their ability to utilize HMOs and to develop cross-feeding relationships with butyrate-producing bacteria, such as those belonging to the phylum Firmicutes [11,12]. In particular, *Bifidobacterium longum* subsp. *infantis* (*B. infantis*) contains a complement of genes that support utilization of both fucosylated and sialylated HMOs [13]. Metabolism of HMO by *B. infantis* upregulates solute-binding proteins with an affinity for HMOs genes and glycosidases, which further aids in its ability to breakdown HMOs, specifically 2’-FL [12,13,14]. Metabolism of 2’-FL by *B. infantis* produces acetate, resulting in cross feeding to other bacteria. 

While *B. infantis* appears to have co-evolved with HMO in a synbiotic relationship, surveys of the fecal microbiota have shown that the relative abundance of *Bifidobacterium*, particularly *B. infantis,* in the feces of breastfed infants in the US [15] is lower than that of infants in the developing world [16,17]. Thus, administration of *B. infantis* to breastfed infants has gained popularity as a commercially available probiotic due to its associated benefits [18,19,20]. Among commercially available products is the *B. longum* subsp. *infantis* strain Bi-26 (Bi-26) from the Danisco Global Culture Collection, which was shown to metabolize fucosylated HMOs more rapidly and efficiently than the type strain of *B. infantis* ATCC 15697 [21,22]. Thus, co-administration of 2’-FL and Bi-26 have the potential to confer benefits to formula-fed infants; however, this has not been tested in vivo. 

Thus, the goal of this study was to evaluate the effects of individual and synbiotic supplemental 2’-FL and Bi-26 to formula on body weight gain, organ growth and gastrointestinal development and function in the piglet model. The pig is a widely accepted preclinical model due to more shared similarities in gut anatomy and physiology to the human infant than do rodent models [23,24,25]. We hypothesized that 2’-FL and Bi-26 alone or in combination would be well tolerated and support normal piglet growth, and aimed to investigate any synergy between 2’-FL and Bi-26. Based on previous evidence [8,9,10], we further hypothesized that 2’-FL would promote intestinal development in vivo.

## 2. Materials and Methods

### 2.1. Study Design

The Institutional Animal Care and Use Committee at the University of Illinois approved all animal procedures (Protocol 17286). Naturally farrowed piglets from a commercial swine herd remained with the sows until postnatal day 2 (PND 2) before being transported to a specialized neonatal pig rearing system at the Piglet Nutrition and Cognition Laboratory (PNCL) on the University of Illinois campus. A total for 63 male piglets were randomly assigned to treatment groups by equalizing initial body weight and genetics (i.e., litter of origin) across treatments, such that an equal number of pigs from each litter was assigned to each treatment group in every cohort. The treatment structure included a 2 × 2 factorial arrangement of dietary [commercial milk replacer ad libitum without (CON) or with 1.0 g/L 2’-FL (FL)] and probiotic treatments. Within each dietary treatment, piglets were further randomized to receive either glycerol solution alone (BI) or Bi-26 (10^9^ CFU) (FLBI) orally once daily. The experiment was conducted in five cohorts. Piglets of only one sex (male) were chosen to reduce any source of variation; although it was recently demonstrated that body and organ growth does not differ for pigs in the age range used in this study [26].

All piglets were monitored continuously using home-cage cameras that permitted asynchronous viewing of animal health and well-being throughout the study. Additionally, animal caretakers who were blinded to experimental treatment identity performed twice-daily in-person health checks (0800 and 1600 h) that included the following outcomes for individual pigs: (1) fecal consistency (1 = solid; 2 = semisolid; 3 = loose; 4 = watery), (2) body condition (5-point scale), (3) visual inspection of feeding system and milk bowl, (4) piglet responsiveness upon approach and general demeanor (lethargy; yes/no), and (5) evidence of piglet vomiting (yes/no). In total, 10 piglets were removed from the study due to failure to thrive unrelated to experimental treatments. The number removed per cohort was: Cohort 1 (*n* = 3); Cohort 2 (*n* = 0); Cohort 3 (*n* = 2); Cohort 4 (*n* = 3); and Cohort 5 (*n* = 2). The final number per treatment group was: CON (*n* = 12); BI (*n* = 14); FL (*n* = 15); and FLBI (*n* = 12). 

### 2.2. Experimental Diets

Two milk replacer diets (CON and FL) were manufactured in powder form by TestDiet (Purina Mills, St. Louis, MO, USA). The composition was identical to ProNurse (Land O’Lakes, North Arden Hills, MN, USA) with the exception that the CON diet had added 0.532% lactose to match the 2’-FL content in the test diet (Table 1). The 2’-FL was supplied as Care4U 2’-fucosyllactose (International Flavors & Fragrances; New York, NY, USA). Diets were shipped to the University of Illinois as color-coded bags, so that researchers working with the piglets were blinded to the dietary treatment. Throughout the study, experimental milk replacer powders were reconstituted fresh each day with 200 g of dry power being added to 800 mL of tap water. Analyzed values are presented for the experimental dietary treatments both in the powder form and after the powder was reconstituted into a liquid form (Table 1). Milk was dispensed into clean reservoirs and automatically delivered to piglets using a pump beginning at 10 am each day and ending at 6 am the following day (i.e., ad libitum access to treatment diets over a 20 h feeding period). Milk disappearance was calculated per pig based on the initial and final reservoir weights before and after the daily feeding cycle, respectively. Based on milk disappearance quantified the preceding day, a sufficient volume of reconstituted milk replacer treatment was dispensed into individual reservoirs per pig to ensure ad libitum access to milk each day.

### 2.3. Probiotic Treatment

Piglets in the CON and FL diet groups were further randomized to receive probiotic or no probiotic, resulting in four experimental treatment groups (CON, BI, FL, FLBI). Aliquots of Bi-26 (Bi-26 50B 100 GM, FloraFIT^®^ Probiotics; Danisco USA Inc., Madison, WI, USA) at the dose of 10^9^ CFU/pig/d was solubilized prior to the start of the study in bacterial glycerol stock (12.1% glycerol) and frozen at −80 °C until administration. From PND 2-PND 12, the probiotic dose was thawed and orally administered using a syringe prior to the first feed of the day. Piglets not receiving probiotic treatment received an equal dose of glycerol solution. From PND 13 to PND 33 or 34, piglets received either probiotic treatment or glycerol solution mixed with approximately 10 mL of milk in their bowl prior to the first feeding of the day. The study duration was based on known changes in brain structure and development from previous studies [27].

### 2.4. Sample Collection

Each morning, piglets were weighed, and any remaining formula was measured. On PND 34 or 35, piglets were sedated with an intramuscular injection of Telazol^®^ (Tiletamine HCl and Zolazepam HCl, 3.5 mg/kg BW each, Pfizer Animal Health, Fort Dodge, IA, USA). Piglets were then euthanized by an intracardiac injection of sodium pentobarbital (72 mg/kg BW; Euthasol, by Virbac, Westlake, TX, USA). The brain and liver were removed and weighed. The small intestine was cut at 10% and 85% from the proximal end to give 3 segments corresponding to the duodenum, jejunum and ileum, respectively. A 25 cm section of jejunum and ileum were cut longitudinally, and mucosal scrapings were obtained and frozen in liquid nitrogen and stored at −80 °C. In addition, the large intestine was excised, and segments were cut from the most proximal and distal parts corresponding to the ascending colon (AC) and rectum (RC), respectively. Five-centimeter pieces of each intestinal segment were fixed in formalin for 48 h for histomorphological analyses.

### 2.5. Intestine Histomorphology

Formalin-fixed sections of the jejunum, ileum and AC were processed into paraffin blocks, which were sectioned, placed on glass slides and stained with hematoxylin and eosin for the measurement of small intestinal villus length and width, villus area, crypt depth and width, crypt volume, villus-to-crypt ratio, and surface area. Mucosa thickness was measured in AC. Villus area and crypt volume were calculated by multiplying width by villus height or crypt depth, respectively. Tissue processing and staining, and all measurements were performed by Dr. Fred Hoerr, a board-certified veterinary pathologist (Veterinary Diagnostic Pathology, LLC., Fort Valley, VA, USA). 

### 2.6. Disaccharidase Activities

Intestinal lactase and sucrase activities were measured as previously described [28,29]. Mucosal homogenates were prepared and incubated with 0.6 M lactose (Thermo Fischer Scientific, Hanover Park, IL, USA) or 0.3 M sucrose (Thermo Fisher Scientific, Hanover Park, IL, USA) buffer (dissolved in 0.0625 maleate buffer) for 60 min at 37 °C. The reactions were stopped by adding 2.0% zinc sulfate (Thermo Fisher Scientific, Hanover Park, IL, USA) and 1.8% barium hydroxide (Thermo Fisher Scientific, Hanover Park, IL, USA), and the amount of glucose release was detected by using a glucose oxidase reagent (Thermo Fisher Scientific, Hanover Park, IL, USA). Enzyme activity is expressed as µmoles glucose/minute/g protein. Protein was measured in intestinal homogenates using the Bradford method (Bio-Rad, Hercules, CA, USA).

### 2.7. Dry Matter of Luminal Contents

Dry matter (DM) of AC, cecum (CE) and RC contents were assessed on the samples according to the AOAC method [30]. Initial (~4 g) and final weights of contents were recorded after drying and % DM was calculated.

### 2.8. DNA Extraction from Luminal Contents

Microbial whole genomic DNA was isolated from AC and DC contents by the QIAamp DNA Stool Mini Kit (Qiagen, Hilden, Germany) in combination with the FastPrep-24 bead beating system (MP Biomedicals, Santa Ana, CA, USA) as previously described [31]. DNA concentration was determined with a Qubit 3.0 Fluorometer (Life Technologies, Carlsbad, CA, USA) and DNA quality was confirmed with agarose electrophoresis.

### 2.9. qPCR for Bifidobacterium spp. and B. infantis

Absolute abundances of *Bifidobacterium* spp. and *B. infantis* were quantified in AC and RC with the use of primers specific for *Bifidobacterium* spp. [32] and *B. infantis* [33], respectively. Real-time qPCR was performed by QuantStudio 7 Real-Time PCR systems (Thermo Fisher Scientific, Hanover Park, IL, USA) using SYBR Green Master Mix (Applied Biosystems, Foster City, CA, USA). All experiments were performed in triplicated with a total reaction volume of 10 μL, containing 5 μL of 2 × Power SYBR Green Master Mix, 0.5 μM of each primer, 1 μL (1 mg/mL) bovine serum albumin (BSA), 1 μL of DEPC water and 2 μL of DNA template diluted 1:30 or 1:15. The cycling conditions were as follows: 50 °C for 2 min, 90 °C for 10 minutes followed by 40 cycles of 95 °C for 15 s, 60 °C or 55 °C for 20 s for *Bifidobacterium* spp. or *B. infantis, respectively*, and 72 °C for 45 s. Following amplification, a dissociation step was included to assess the melting profile of amplified products. Standard curves (10–10^8^ 16S ribosomal RNA (rRNA) gene copies/reaction) were generated using purified 2.1 TOPO-TA plasmids containing full length 16S rRNA gene of *B. infantis* (Thermo Fisher Scientific, Hanover Park, IL, USA). Data were processed with QuantStudio Real-Time PCR Software V1.3 (Thermo Fisher Scientific, Hanover Park, IL, USA) and expressed as log^10^ 16S rRNA gene copies.

### 2.10. Statistical Analysis

Statistical analyses were performed using SAS version 9.4 (SAS Institute Inc., Cary, NC, USA) using the PROC MIXED procedure. BW and formula intake were analyzed using a repeated-measure analysis of variance (ANOVA). Organ weights, disaccharidase activity, intestine histomorphology, and qPCR abundance data were analyzed using a 2-way ANOVA. Both statistical models included a nested model for random effects with replicate nested within sow. Both models included fixed effects of diet, probiotic and the interaction between diet and probiotic with Dunnett’s post hoc test to compare statistical difference between the control group and experimental treatment groups. The occurrence of *B. infantis* among treatments was analyzed by Fisher’s exact test using the PROC FREQ procedure in SAS. If samples fell outside of ± three standard deviations from the mean, they were excluded as outliers. The following data were transformed to meet the assumptions of normality and confirmed post hoc: jejunal and ileal disaccharidase activity and abundance of *Bifidobacterium* spp. and *B. infantis*. Significance was set at *p* < 0.05 and trends are reported as *p* < 0.10. Data are presented as means ± standard error of the mean (SEM) unless otherwise stated.

## 3. Results

### 3.1. Tolerance, Weight Gain, Organ Weights and Intestinal Length and Weight

**Figure 1:**
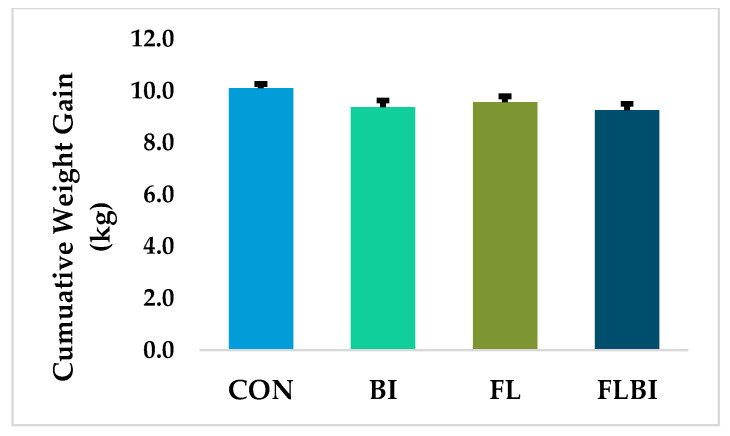
Total body weight gain of piglets. Total body weight gain between PND 2 from PND 33. Data are expressed as means ± SEM. *n* = 12–15. Experimental treatment tended (*p* = 0.075) to impact total weight gain. Abbreviations: BI, control diet + 10^9^ CFU Bi-26/day; CON, control diet; FL, control formula + 1.0 g/L 2’-FL; FLBI, control formula + 1.0 g/L 2’-FL + 10^9^ CFU Bi-26/day; PND, postnatal day; SEM, standard error of the mean.

Experimental diets and probiotic treatments were successfully administered, and Bi-26 stored frozen in glycerol stock remained viable throughout the study (Table S1). There was no significant treatment differences in any of the assessed behavioral or general demeanor outcomes and piglets in all groups demonstrated a typical pattern of body weight gain for artificially reared animals in the PNCL facility [26]. Although animals were allowed ad libitum access to the diets, relative formula intake over the course of the study did not differ between treatments, with an average of 316.9 ± 11.6 mL/kg BW/d (*p* = 0.419). Daily weight gain was affected by probiotic and diet, but without interactive effect (Figure S1). Total weight gain from PND2 to PND33 showed a statistical trend (*p* = 0.075) on the interactive effects of diet and probiotic, with CON piglets showing the greatest total body weight gain (Figure 1). Brain weights adjusted per kg BW tended (*p* = 0.060) to differ, but neither liver weight or small or large intestinal weight or length differed based on absolute or adjusted values (Table S2).

### 3.2. Intestine Histomorphology and Disaccharidase Activity

Jejunal morphology was similar across treatments (Table S3). In the ileum, there were no effects of treatment on villus morphology (Table 2), but crypt width (*p* = 0.071) and crypt volume (*p* = 0.069) were numerically greater in piglets fed the FL diet (Table 2) and the interaction between diet and probiotic was statistically significant at trend level. Crypt depth showed a main effect of probiotic, with shorter crypts in the BI treatment group than the CON and FL groups (*p* = 0.006), as well as a main effect of diet, with crypt depth in the FL group being less than CON. (Table 2). Mucosa thickness in the AC was similar across all treatment groups (Table S4). 

Lactase and activity in jejunum and ileum were similar across all treatments (Table S5), but the addition of 2’-FL to the diet resulted in a significant increase in sucrase activity (Table 3).

### 3.3. Dry Matter of Luminal Contents and qPCR for Bifidobacterium spp. and B. infantis

Dry matter was significantly higher in RC than CE and AC (*p* < 0.001) (Figure S2). However, there was no differences in dry matter due to an experimental treatment (*p* = 0.752). The absolute quantity of *Bifidobacterium* spp. was similar across treatments in AC and RC contents (Table S6). *B. infantis* was detected in ≥50% of BI and FLBI piglets, but in none of the CON or FL piglets (Table 4). The percent of AC and RC samples from piglets in the BI and FLBI groups with detectable *B. infantis* was similar (*p* = 0.172). In both the AC and RC, *B. infantis* abundance was greater in the BI and FLBI than CON or FL, but there were no differences in the abundance of *B. infantis* between the BI and FLBI groups (Table 4).

## 4. Discussion

HMOs are complex glycans [5] that withstand digestion of the upper gastrointestinal tract [6,7] to act as a prebiotic in the lower gastrointestinal tract [11,12], with physiological benefits for the infant [1,2,3,34]. HMO are proposed to influence intestinal development [8,9,10], as well as microbiota composition by promoting growth of *Bifidobacterium* species, including *B. infantis* [11,12,13]. Infant formulas that contain prebiotics, such as galactooligosaccharides (GOS) and fructooligosaccharides, support *Bifidobacterium* growth are currently commercially available [34,35]. However, the chemical structures of commercial prebiotics added to formula and HMOs differ substantially, and it has been suggested that HMOs may be better suited to the infant’s developing gastrointestinal system [36]. Piglets and infants share similarities in gastrointestinal anatomy and metabolism [23,24,25], and piglets display a closer similarity in gut microbiota composition to humans than do rodents [37]. Thus, they serve as a relevant preclinical model to study prebiotics and probiotics and have been used extensively by our group [38,39,40] and others [41,42] to investigate the biological effects of individual HMOs and *B. infantis* [43]. The present study is the first to compare the individual and combined effects of HMO and *B. infantis* in the piglet model. 

Supplementation of 2’-FL at 1 g/L and Bi-26 at 10^9^ CFU/d were well tolerated and supported normal growth throughout the study. CON piglets tended to gain more weight than FLBI and BI had lower feed efficiency than CON. Data on the effect of HMO on weight gain are conflicting. In suckling rat pups, weight gain was greater by the end of the study in those who received 2’-FL (0.2 g/100 g body weight) vs. the control group, who received a gavage of only mineral water [44]. This dose was estimated to be similar to the daily consumption of 2’-FL of human infants [45]. Data from human infants differ depending on whether the HMO was supplemented to infant formula [46,47] or studies examined associations between HMO concentrations in milk and infant weight gain [48,49,50]. In randomized controlled trials, infants fed formula containing 2’-FL alone (0.2 or 1.0 g/L) [46] or with lacto-N-neotetraose (LNnT) (1.0 g/L 2’-FL and 0.5 g/L LNnT) [47] had similar growth to infants fed formula alone. In contrast, a study of breastfed infants demonstrated that the abundance of two fucosylated HMOs, 2’-FL and difucosyl-lactose (DFlac), were associated with increased weight velocity at 5 months, whereas LNnT was negatively associated with height-for-age Z-scores [48]. In contrast, concentrations of other HMOs, including lacto-N-fucopentaose I and LNnT, were positively associated with weight and body composition at 6-months of age in breastfed infants; however, there were no associations with 2’-FL [50]. Lastly, a meta-analysis of the modulation of the microbiota and weight gain showed that probiotic supplementation in infants has little effect on weight gain [51]. The present study showed that supplementation with 2’-FL and Bi-26 individually or together lowered daily weight gain and together tended to lower total body weight gain compared to the CON formula. However, it should be noted that piglets demonstrated a typical growth pattern for piglets raised in the artificial rearing system in our facility [26] and total weight gain in the treatment groups was only ~5% lower than control; thus, it is likely that they difference body weight would not be of clinical relevance. 

To assess the impact of 2’-FL and/or Bi-26 supplementation on intestine function and development, intestinal morphology and disaccharidase activities were assessed. Previous research has shown that pooled HMOs or 2’-FL alone at biologically relevant concentrations inhibited intestinal cell proliferation and increased alkaline phosphatase activity, a marker of cellular differentiation, in vitro [8,9]. In suckling rats, daily oral administration of 0.2 g 2’-FL/100 g BW increased jejunal villus height and area on day 8 postpartum compared to suckling rat pups not administered 2’-FL [44]. In the current study, 1 g/L 2’-FL had no effect on jejunal or ileal villus height, but ileal crypt width and area tended to be greater in piglets fed 2’-FL. Our study is in agreement with previous work which evaluated formula supplementation with 4 g/L HMOs, of which 40% was 2’-FL, showed no impact of HMO on pig jejunal or ileal morphology following rotavirus infection [38]. An increase in ileal sucrase activity in response to dietary 2’FLwas observed, which was also shown in vitro [8]. However, two other pig studies with HMO supplementation did not detect any effect on small intestinal disaccharidase activity [39,41]. Thus, more studies are needed to understand the impact of 2’-FL on sucrase activity in the developing intestine. In terms of Bi-26, the only statistically significant effect was a reduction in ileal crypt depth. Shorter crypts could indicate lower proliferation, however, no differences in villus height were observed. Taken together, these findings suggest that 2’-FL and Bi-26 had little impact on intestinal development in the piglet. 

Lastly, we analyzed the absolute abundance and detectability of *B. infantis* in the AC and RC. A previous study from our lab has shown successful colonization of *B. infantis* ATCC 15697 at 3.0 × 10^9^ per day in a piglet model [43]. Herein, the abundance (Log_10_ copies/g feces) of the *Bifidobacterium* genera was similar to previous studies investigating the impact of diet on the piglet microbiota [39,43,52] and were unaffected by treatment with Bi-26 and 2’-FL. However, primers designed specifically for *B. infantis* demonstrated that *B. infantis* was only detected in the AC and RC of piglets in the BI and FLBI groups, verifying that the correct treatments were administered throughout the study. Although only BI and FLBI piglets had detectible levels of *B. infantis*, it was not detected in samples from all piglets. This is likely due to the fact that the piglets were administered their last dose of *B. infantis* on the morning of the day prior to sample collection. Thus, *B. infantis* had persisted for more than 24 h in some, but not all piglets. In infants, the *B. infantis* EVC001 strain at 1.8 × 10^10^ CFU have been shown to colonize the gut even after supplementation stops, as long as the infants continued to be breastfed [19]. The current study used a different strain of *B. infantis* (Bi-26) than the human study and those infants were receiving a complex mixture of HMO through breastfeeding [19]. The Bi-26 strain of *B. infantis* metabolized fucosylated HMOs more rapidly and efficiently than the strain type *B. infantis* ATCC 15697 in vitro [21,22], however, there were no differences in the presence or abundance (log10 counts/g feces) of *B. infantis* in either the AC or RC of the BI and FLBI groups. This finding suggests that in vitro evidence may not translate to a synbiotic effect of 2’-FL when co-administered with Bi-26 in vivo. A similar finding was observed by Musilova and colleagues, where the synbiotic effects of HMO and *B. infantis* that were observed in vitro, were not seen in humanized mice [53]. Although the native piglet microbiota has a lower abundance of *Bifidobacterium* than the human infant [39,43,52], this study, and our previous work [43], demonstrate that the piglet intestine will accept the probiotic administration of *B. infantis* without adverse effect. Thus, the piglet is a robust model in which to study the bioactivity of probiotics and host-microbe interactions [54].

## 5. Conclusions

The American Academy of Pediatrics recommends exclusive breastfeeding for the first 6 months of life [55], however; only a quarter of infants in the US meet this goal [56]. Thus, there is a desire to improve the composition of infant formula in order to narrow the differences in microbial diversity, gut health and immune outcomes between breast and formula-fed infants, and oligosaccharides and *Bifidobacterium* have been identified as prime candidates [57,58,59]. In summary, this study demonstrates that the addition of 2’-FL and/or Bi-26 is well tolerated in piglets. Piglets administered 2’-FL and/or Bi-26 showed a normal growth pattern and did not demonstrate any adverse effects on overall intestine, liver or brain growth. Region-specific effects of supplementation were observed within the intestine. 2’-FL tended to increase crypt depth and increased sucrase activity in the ileum, whereas Bi-26 decreased ileal crypt depth and increased lactase activity in the jejunum. Thus, the addition of 2’-FL and/or Bi-26 could provide the formula-fed infant the benefits of the bioactive components that infant formulas typically lack; such a formula should be compared to breastfeeding as a benchmark.

## Figures and Tables

**Table 1:** Nutritional composition of experimental pig milk replacer treatments ^1^.

	Dietary Treatment ^2^			
	CON	FL	CON	FL
Dietary Form	Powder	Powder	Liquid	Liquid
Units	g/100 g DM	g/100 g DM	g/L	g/L
Dry matter	97.22	97.21	194.44	194.42
Organic matter	90.75	90.84	176.45	176.611
Lactose	40.05	39.16	77.88	76.13
2’-fucosyllactose	ND	0.63	ND	1.22
Ash	9.25	9.16	17.99	17.81
Phosphorus	0.73	0.72	1.43	1.40
Calcium	0.95	0.92	1.85	1.79
Acid-hydrolyzed fat	26.29	26.32	51.12	51.17
Crude protein	23.71	23.95	46.10	46.56
Total amino acids	23.56	24.72	45.81	48.06
Essential amino acids				
Arginine	0.66	0.68	1.28	1.32
Histidine	0.47	0.48	0.91	0.93
Isoleucine	1.42	1.44	2.76	2.80
Leucine	2.45	2.49	4.76	4.84
Lysine	2.21	3.10	4.30	6.03
Methionine	0.42	0.45	0.82	0.87
Phenylalanine	0.89	0.91	1.73	1.77
Threonine	1.48	1.50	2.88	2.92
Tryptophan	0.43	0.44	0.84	0.86
Valine	1.37	1.39	2.66	2.70
Non-essential amino acids				
Alanine	1.11	1.12	2.16	2.18
Aspartic acid ^3^	2.39	2.42	4.65	4.70
Cyst(e)ine ^4^	0.50	0.51	0.97	0.99
Glutamic acid ^5^	3.92	3.94	7.62	7.66
Glycine	0.50	0.50	0.97	0.97
Proline	1.41	1.40	2.74	2.72
Serine	1.07	1.10	2.08	2.14
Tyrosine	0.68	0.70	1.32	1.36

^1^ Abbreviations: CON, control treatment; DM, dry matter; FL, 2’-fucosyllactose treatment; ND, not detected; ^2^ Dietary treatment powders were produced starting with a commercially available milk replacer (ProNurse Multi-species Milk Replacer; Land O’Lakes, Arden Hills, MN, USA) and dry-blended by TestDiet (St. Louis, MO, USA). Both dietary treatments reserved 0.532% of the formulation for either lactose (CON) or 2’-fucosyllactose (FL; supplied as Care4U 2’-fucosyllactose; International Flavors & Fragrances; New York, NY, USA); ^3^ Analysis does not distinguish between aspartic acid and asparagine, so the reported value is summative; ^4^ Analysis does not distinguish between the reduced (cysteine) plus oxidized (cystine; disulfide) forms, so the reported value is summative; ^5^ Analysis does not distinguish between glutamic acid and glutamine, so the reported value is summative.

**Table 2:** Ileum Histomorphology.

						*p*-Value	
	CON	BI	FL	FLBI	Diet	Probiotic	Interaction
Villus length (μm)	529 ± 25.6	555 ± 34.4	605 ± 39.4	542 ± 37.5	0.9371	0.3502	0.5789
Villus width (μm)	127 ± 5.5	115 ± 4.9	127 ± 4.5	124 ± 3.4	0.3234	0.1300	0.2957
Villus area (μm^2^)	65.5 ± 3.7	64.8 ± 6.6	76 ± 4.9	67.4 ± 5.3	0.2564	0.4062	0.4613
Crypt width (μm)	38.4 ± 0.8	41.6 ± 1.4	42.3 ± 1.1	41.3 ± 1.7	0.2285	0.5432	0.0709
Crypt depth (μm)	168 ± 7.0	145 ± 6.0	184 ± 7.5	159 ± 4.3	0.0402	0.0006	0.6804
Crypt volume (μm^3^)	5853 ± 320	5728 ± 362	7080 ± 443	5961 ± 366	0.0962	0.0659	0.0686
Surface area (μm^2^)	205 ± 28	204 ± 27.3	238 ± 15.2	212 ± 17.2	0.2499	0.4324	0.4893
Villus-to-crypt ratio	3.6 ± 0.3	3.6 ± 0.3	3.2 ± 0.2	3.5 ± 0.2	0.3483	0.5480	0.5369

Data are expressed as means ± SEM. *n* = 9–12. Abbreviations: BI, control diet + 10^9^ CFU Bi-26/day; CON, control diet; FL, control formula + 1.0 g/L 2’-FL; FLBI, control formula +1.0 g/L 2’-FL +10^9^ CFU Bi-26/day; ILE, ileum; PND, postnatal day; SEM, standard error of the mean.

**Table 3:** Jejunum and Ileum Sucrase Activity.

					*p*-Value		
	CON	BI	FL	FLBI	Diet	Probiotic	Interaction
Jejunum	25.5 ± 0.5	22.3 ± 3.5	23.3 ± 2.4	20.6 ± 3.9	0.6302	0.3396	0.6227
Ileum	14.1 ± 1.7	13.9 ± 2.0	21.6 ± 3.6	25.0 ± 4.6	0.0116	0.7903	0.5158

Data are expressed as means ± SEM. *n* = 8–12. Abbreviations: BI, control diet + 10^9^ CFU Bi-26/day; CON, control diet; FL, control formula + 1.0 g/L 2’-FL; FLBI, control formula + 1.0 g/L 2’-FL + 10^9^ CFU Bi-26/day; ILE, ileum; PND, postnatal day; SEM, standard error of the mean.

**Table 4:** Presence and Absolute Abundance of Gene Copies of *B. infantis* in Ascending Colon and Recal Contents.

	Presence		Abundance (No Imputations) *	
	Positive/Total of Animal (%)		Log_10_ Copies/g Feces	
	Ascending Colon Contents	Rectal Contents	Ascending Colon Contents	Rectal Contents
CON	0/10 (0) ^a^	0/12 (0) ^a^	BLD	BLD
BI	6/12 (50) ^b^	8/13 (62) ^b^	7.58 ± 0.34	8.03 ± 0.13
FL	0/15 (0) ^a^	0/15 (0) ^a^	BLD	BLD
FLBI	9/12 (75) ^b^	8/12 (67) ^b^	7.46 ± 0.11	7.40 ± 0.38

Data are expressed as means ± SEM. *n* = 10–15; different letter superscripts indicates values within a column differ by treatment (*p* < 0.05) by Fisher’s Exact Test; * Values for samples that had detectable levels of *B. infantis*; Abbreviations: BLD, below level of detection; BI, control diet + 10^9^ CFU Bi-26/day; CON, control diet; FL, control formula + 1.0 g/L 2’-FL; FLBI, control formula + 1.0 g/L 2’-FL + 10^9^ CFU Bi-26/day; SEM, standard error of the mean.

## Data Availability

Data will be available upon request.

## Supplementary Materials

The following are available online at https://www.mdpi.com/article/10.3390/nu14010199/s1, Figure S1: Daily body weight gain, Figure S2: Fecal dry matter; Table S1: Viability of Bi-26 stock; Table S2: Absolute and relative organ lengths and weights. Table S3 Jejunum histomorphology; Table S4: Ascending colon histomorphology; Table S5: jejunum and ileum lactase activity; Table S6: Abundance of *B**ifidobacterium.*
