# Supplementary material for: Evaluation of 2’-Fucosyllactose and Bifidobacterium longum Subspecies infantis on Growth, Organ Weights, and Intestinal Development of Piglets
Source: Nutrients. 2021 Dec 31;14(1):199. doi: 10.3390/nu14010199 (PMC8747721; doi:10.3390/nu14010199)
Supplement: Supplementary file 1 [file nutrients-14-00199-s001.zip › nutrients-1491913-SI.pdf]

Table S1. Viability of frozen Bi-26 stock.

Cohort # (PND of Cohort)	Date of Culture	Calculated Dose Administered (CFU)
C1 (PND29)	8/23/2019	1.4×10^9
C2 (PND33)	9/10/2019	1.4×10^9
C4 (PND33)	9/24/2019	1.1×10^9
C5 (before piglet arrival)	1/24/2020	1.7×10^9

Abbreviations: Bi-26, *Bifidobacterium longum* subsp. *infantis;* CFU, colon-forming unit; PND, postnatal day.

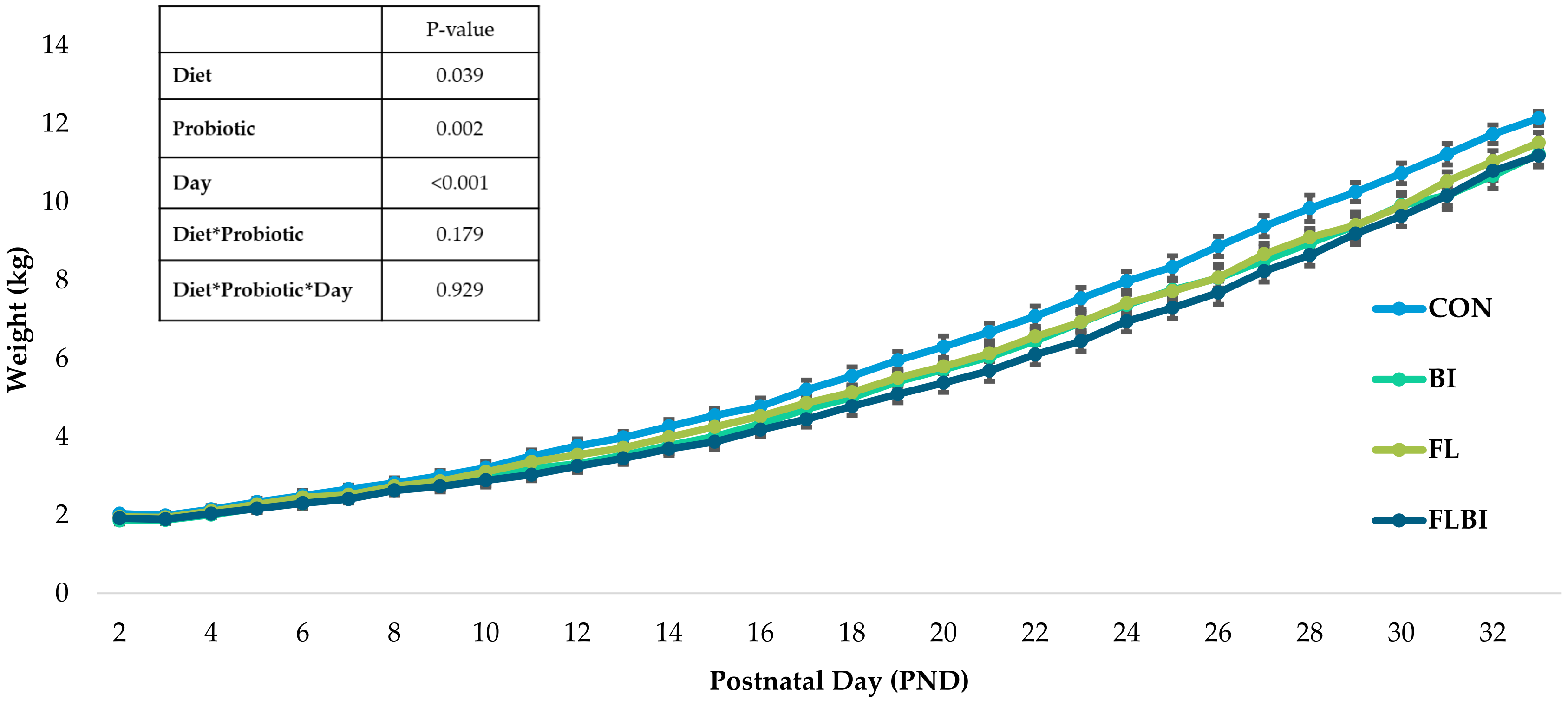

	P-value
**Diet**	0.039
**Probiotic**	0.002
**Day**	<0.001
**Diet*Probiotic**	0.179
**Diet*Probiotic*Day**	0.929

Figure S1. Body weight gain of piglets**.** Body weights were measured daily between PND 2 and PND 34/35. Diet, probiotic and day significantly affected daily body weight gain, but there was no interactive effect between diet and probiotic to lower daily body weight gain. Data are expressed as means ± SEM. n=12-15. Abbreviations: BI, control diet + 10^9 CFU Bi-26/day; CON, control diet; FL, control formula + 1.0 g/L 2'-FL; FLBI, control formula +1.0 g/L 2'-FL +10^9 CFU Bi-26/day; PND, postnatal day; SEM, standard error of the mean.

Table S2. Absolute and Relative Organ Lengths and Weights. Organ lengths (cm) and weights (g) were adjusted per kg BW at sacrifice on PND 34/35.

	CON	BI	FL	FLBI	P-value		
					**Diet**	**Probiotic**	**Interaction**
Small intestine length (cm)	1415 ± 33	1394 ± 27	1351 ± 25	1397 ± 38	0.332	0.740	0.331
Small intestine length (cm/kg BW)	116 ± 1.9	124 ± 4.4	117 ± 3.4	122 ± 4.2	0.818	0.131	0.241
Small intestine weight (g)	487 ± 16	487 ± 15	484 ± 19	523 ± 38.5	0.367	0.202	0.446
Small intestine weight (g/kg BW)	40 ± 1.5	43.2 ± 1.2	41.9 ± 1.1	43.7 ± 2.2	0.553	0.122	0.232
Large intestine length (cm)	211 ± 5.4	198 ± 7.3	218 ± 4.5	214 ± 4.7	0.242	0.153	0.653
Large intestine length (cm/kg BW)	17.4 ± 0.6	18.5 ± 0.6	18.9 ± 0.5	18.7 ± 0.7	0.371	0.335	0.134
Liver weight (g)	359 ± 7.8	331 ± 7.2	367 ± 11	351 ± 11	0.157	0.027	0.559
Liver weight (g/kg BW)	29.3 ± 0.6	29.4 ± 0.6	31.6 ± 0.7	29.8 ± 0.9	0.072	0.185	0.161
Brain weight (g)	52.7 ± 1.2	51.3 ± 0.9	52.9 ± 0.8	51.6 ± 1.0	0.932	0.199	0.947
Brain weight (g/kg BW)	4.3 ± 0.1	4.6 ± 0.2	4.6 ± 0.2	4.5 ± 0.1	0.688	0.411	0.060

Data are expressed as means ± SEM. n=11-15.

Abbreviations: BI, control diet + 10^9 CFU Bi-26/day; BW, body weight; CON, control diet; FL, control formula + 1.0 g/L 2'-FL; FLBI, control formula +1.0 g/L 2'-FL +10^9 CFU Bi-26/day ; PND, postnatal day; SEM, standard error of the mean.

Table S3. Jejunum Histomorphology.

					P-value		
	**CON**	**BI**	**FL**	**FLBI**	**Diet**	**Probiotic**	**Interaction**
Villus length (µm)	612 ± 19.8	598 ± 34	621 ± 18.1	576 ± 35.4	0.811	0.295	0.576
Villus width (µm)	124 ± 4.4	120 ± 4.3	124 ± 3.9	116 ± 4.1	0.567	0.135	0.780
Villus area ($µm^2$)	75.7 ± 2.8	72.4 ± 6.1	76.9 ± 3.9	67.5 ± 5.3	0.695	0.194	0.524
Crypt width (µm)	45.5 ± 1.2	45 ± 0.5	45.5 ± 1.4	45.6 ± 1.1	0.781	0.887	0.779
Crypt depth (µm)	193 ± 10.1	188 ± 9.8	200 ± 9.4	189 ± 7.5	0.670	0.390	0.784
Crypt volume ($µm^3$)	8747 ± 471	8472 ± 519	9118 ± 567	8650 ± 446	0.593	0.497	0.850
Surface area ($µm^2$)	237 ± 8.3	227 ± 19	241 ± 12.3	212 ± 16.9	0.724	0.203	0.508
Villus-to-crypt ratio	3.3 ± 0.2	3.3 ± 0.2	3.0 ± 0.2	3.1 ± 0.2	0.353	0.775	0.852

Data are expressed as means ± SEM. n=10-12.

Abbreviations: BI, control diet + 10^9 CFU Bi-26/day; CON, control diet; FL, control formula + 1.0 g/L 2'-FL; FLBI, control formula +1.0 g/L 2'-FL +10^9 CFU Bi-26/day; PND, postnatal day; SEM, standard error of the mean. .

Table S4. Ascending Colon Histomorphology.

	CON	BI	FL	FLBI	P-value		
					**Diet**	**Probiotic**	**Interaction**
Mucosa Width (μm)	412 ± 19.4	376 ± 12.2	408 ± 8.7	386 ± 17.9	0.886	0.068	0.621

Data are expressed as means ± SEM. n=10-12.
Abbreviations: BI, control diet + 10^9 CFU Bi-26/day; CON, control diet; FL, control formula + 1.0 g/L 2'-FL; FLBI, control formula +1.0 g/L 2'-FL +10^9 CFU Bi-26/day ; PND, postnatal day; SEM, standard error of the mean.

Table S5. Jejunum and Ileum Lactase Activity (μmoles glucose/minute/g protein).

					P-value		
	**CON**	**BI**	**FL**	**FLBI**	**Diet**	**Probiotic**	**Interaction**
Jejunum	14.9 ± 1.7	19.4 ± 2.9	11.6 ± 1.9	16.5 ± 2.4	0.107	0.035	0.601
Ileum	3.0 ± 0.4	4.8 ± 1.1	3.0 ± 0.5	4.11 ± 1.1	0.839	0.138	0.609

Data are expressed as means ± SEM. n=10-12.
Abbreviations: BI, control diet + 10^9 CFU Bi-26/day; CON, control diet; FL, control formula + 1.0 g/L 2'-FL; FLBI, control formula +1.0 g/L 2'-FL +10^9 CFU Bi-26/day; SEM, standard error of the mean.

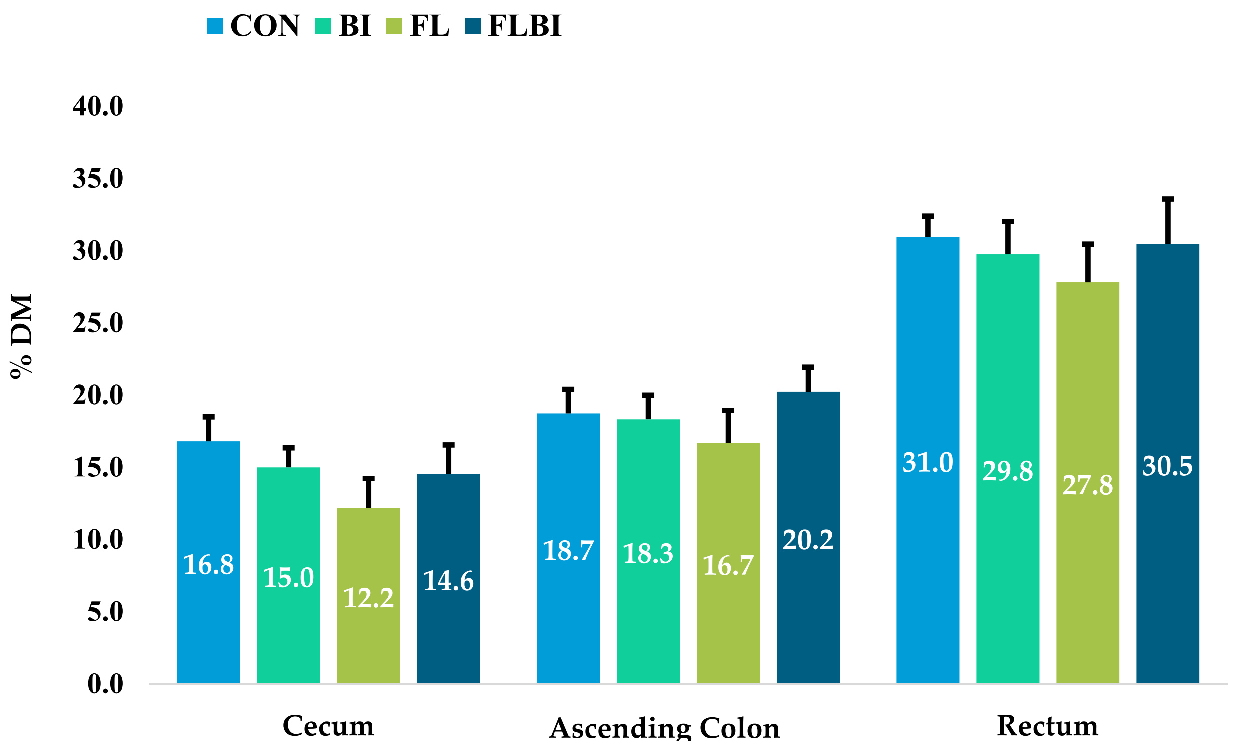

Figure S2. Dry Matter Content of Cecum, Ascending and Rectal Contents. Dry matter content was unaffected by diet or probiotic, but was higher ($p<0.001$) in the rectal contents than the cecum or ascending colon contents. Data are expressed as means ± SEM. n=10-15. Abbreviations: BI, control diet + 10^9 CFU Bi-26/day; CON, control diet; DM, dry matter; FL, control formula + 1.0 g/L 2'-FL; FLBI, control formula +1.0 g/L 2'-FL +10^9 CFU Bi-26/day; SEM, standard error of the mean.

Table S6. Abundance of gene copies of *Bifdobacterium* genera (log_{10} copies).

					P-values		
	CON	BI	FL	FLBI	Diet	Probiotic	Interaction
Ascending colon	7.73 ± 0.19	7.81 ± 0.24	8.04 ± 0.23	7.96 ± 0.22	0.335	0.996	0.796
Rectum	7.77 ± 0.20	8.08 ± 0.18	8.17 ± 0.24	7.93 ± 0.20	0.559	0.870	0.211

Data are expressed as means ± SEM. n=10-15.

Abbreviations: BI, control diet + 10^9 CFU Bi-26/day; CON, control diet; FL, control formula + 1.0 g/L 2'-FL; FLBI, control formula +1.0 g/L 2'-FL +10^9 CFU Bi-26/day ; SEM, standard error of the mean
